# Eccentric exercise 24 h prior to hypobaric decompression increases decompression strain

**DOI:** 10.1007/s00421-023-05214-3

**Published:** 2023-05-04

**Authors:** Frode Gottschalk, Ola Eiken, Antonis Elia, Mikael Gennser

**Affiliations:** 1 Division of Environmental Physiology, Swedish Aerospace Physiology Center, KTH Royal Institute of Technology, Stockholm, Sweden; 2 Department of Neuroscience, Experimental Traumatology, KI Karolinska Institutet, Stockholm, Sweden

**Keywords:** Decompression sickness, Venous gas emboli, Muscle injury, Delayed-onset muscle soreness

## Abstract

**Purpose:**

Animal studies have shown that recent musculoskeletal injuries increase the risk of decompression sickness (DCS). However, to date no similar experimental study has been performed in humans. The aim was to investigate if exercise-induced muscle damage (EIMD)—as provoked by eccentric work and characterized by reduced strength and delayed-onset muscle soreness (DOMS)—leads to increased formation of venous gas emboli (VGE) during subsequent hypobaric exposure.

**Methods:**

Each subject (*n* = 13) was on two occasions exposed to a simulated altitude of 24,000 ft for 90 min, whilst breathing oxygen. Twenty-four hours prior to one of the altitude exposures, each subject performed 15 min of eccentric arm-crank exercise. Markers of EIMD were reduction in isometric m. biceps brachii strength and DOMS as assessed on the Borg CR10 pain scale. The presence of VGE was measured in the right cardiac ventricle using ultrasound, with measurements performed at rest and after three leg kicks and three arm flexions. The degree of VGE was evaluated using the six-graded Eftedal–Brubakk scale and the Kisman integrated severity score (KISS).

**Results:**

Eccentric exercise induced DOMS (median 6.5), reduced the biceps brachii strength (from 230 ± 62 N to 151 ± 8.8 N) and increased the mean KISS at 24,000 ft, both at rest (from 1.2 ± 2.3 to 6.9 ± 9.2, *p* = *0.01*) and after arm flexions (from 3.8 ± 6.2 to 15.5 ± 17.3, *p* = *0.029*).

**Conclusion:**

EIMD, induced by eccentric work, provokes release of VGE in response to acute decompression.

## Introduction

When the ambient pressure and, thus, the pressure in the body’s tissues is markedly and rapidly reduced, decompression sickness (DCS) can occur. Consequently, DCS may develop in conjunction with diving, flying and extravehicular space activities (“space walks”). Reducing the hydrostatic pressure in a tissue limits its ability to hold physically dissolved gas (mainly nitrogen), and a rapid pressure reduction can therefore lead to gas supersaturation and bubble formation in body tissues and venous blood (venous gas emboli; VGE) (Stepanek 2002; DeHart and Jeffrey 2002). Asymptomatic intravascular bubbles routinely form during decompression and have been detected upon very discrete pressure reductions, e.g. following an ascent from a diving depth of 3.4 msw (Eckenhoff et al. 1990). Absence of detectable VGE has been found to highly correlate with absence of symptoms related to DCS, whereas a large quantity of VGE marks a higher risk of DCS. Under conditions of gas saturation, i.e. when the gas partial pressures in the body tissue compartments are stable and similar to those of the breathing gas, the pressure reduction threshold for provocation of DCS corresponds to an ascent from saturation deeper than 6 msw (Van Liew and Flynn 2005), or to an ascent from sea level to an altitude of about 5500 m, during direct decompression (Webb et al. 2003).

Minimizing the risk of DCS in hyper- or hypobaric decompression can be achieved by restricting the time–pressure exposure profile (i.e. by restricting diving time or time at altitude, rate of ascent and/or diving depth/flying altitude). In addition, DCS-preventive preconditioning techniques have gained considerable attention. Apart from the well-established technique of preoxygenation (Webb and Pilmanis 1999), suggested DCS-preventive measures include physical activity (Gennser et al. 2012), whole-body vibration (Elia et al. 2021), food intake (Theunissen et al. 2015) and medication (Dujić et al. 2006). If, or to what extent, various pre-decompression events might provoke the development of DCS has not been explored to the same extent. Hyperthermic status during the inert gas uptake phase as well as a prolonged period of recumbency and physical inactivity prior to a dive have been found to increase the formation of VGE or risk of DCS (Gerth 2015; Gennser et al. 2018). In addition, exercise during gas uptake at depth is thought to raise the risk of DCS (Jankowski et al. 1997, 2004). Another factor that has been suggested to increase the DCS susceptibility is tissue injury. Thus, studies on cats have shown that acute muscle injuries inflicted prior to hypobaric pressure (and in some cases after decompression from hyperbaric pressure) cause increased bubble formation (Harvey 1945). Contrastingly, smaller injuries (muscle damage) in rats do not appear to increase the risk (Jørgensen et al. 2015, 2013). As regards humans, it has been reported that DCS pain may be overrepresented in body regions that recently have suffered a minor injury (Thompson et al. 1944). Anecdotal observations have suggested increased DCS incidence after long-distance cycling and weightlifting (D. and D. 1993) as well as marked formation of decompression VGE following a skeletal injury (Karlsson et al. 2009). The effects of exercise on the risk of DCS seem somewhat equivocal and the possible influence of exercise-induced muscle damage has not been determined in humans. A non-invasive method to induce microscopic muscle damage in humans (exercise-induced muscle damage: EIMD) is through eccentric muscle actions, when the opposing force exceeds that generated by the muscle, and the contracting muscle is forcibly stretched (Lindstedt et al. 2001).

Thus, high-intensity eccentric muscle work causes substantial overstretching of sarcomeres with signs of structural muscle damage (Mekjavic et al. 2000; Proske and Morgan 2001; Fridén et al. 1981; Newham et al. 1983), triggering a local inflammatory response (Newham 1988; MacIntyre et al. 1995), signified by oedema and soreness, often referred to as delayed-onset muscle soreness (DOMS) (MacIntyre et al. 1995; Jones et al. 1987; Newham et al. 1983; Ebbeling and Clarkson 1989). Typically, maximal voluntary contraction force (MCV) can be reduced by 50% immediately after a bout of high-intensity eccentric exercise and remain significantly reduced 24 and 48 h after the exercise (Clarkson et al. 1992; Prasartwuth et al. 2005). In addition, eccentric exercise may cause disturbances in local perfusion, with impaired flow-mediated dilation (Caldwell et al. 2016; Stacy et al. 2013), damaged capillaries (Stauber et al. 1990) and microvascular hyperpermeability (Hotta et al. 2018).

Individuals who are exposed on a regular basis to substantial changes in ambient pressure, e.g. divers and pilots, are commonly physically active. Hence, it is of interest, not least from a practical viewpoint, to establish, whether, and to what extent, microscopic muscle injuries induced by strenuous physical exercise might increase the risk of developing DCS. Consequently, the aim of this study was to investigate whether eccentric work increases the incidence of venous gas bubbles in connection with hypobaric decompression to 24,000 feet for 90 min.

## Methods

### Subjects

Thirteen healthy men (*n* = 7) and women (*n* = 6) volunteered to participate. Their mean (± standard deviation) age, body mass index and weight were 28.2 (± 1.4) yrs, 25.2 (± 4.2) kg m^−2^, and 83.2 (± 12.1) kg, respectively. None of the subjects had previously experienced DCS. Prior to the first hypobaric exposure, subjects underwent a physical examination of the ear drums, heart, lungs and gross neural functions. Exclusion criteria were: history of cardiorespiratory or metabolic disorders, ongoing infection or other temporary illness, ongoing medication, difficulty in equalizing pressure in the middle ears, ongoing pregnancy. The subjects were informed in detail, both in writing and in speech, about the purpose of the study and the potential risks in participating, prior to giving their written consent. It was also made clear to them that their participation was voluntary and that they had the right to withdraw at any time without any specific reason or explanation. Approval was obtained from the Swedish National Ethics Review Authority (Approval # 2021-05293). The procedures used in this study adhere to the tenets of the Declaration of Helsinki.

### Experimental protocol

**Fig. 1:**
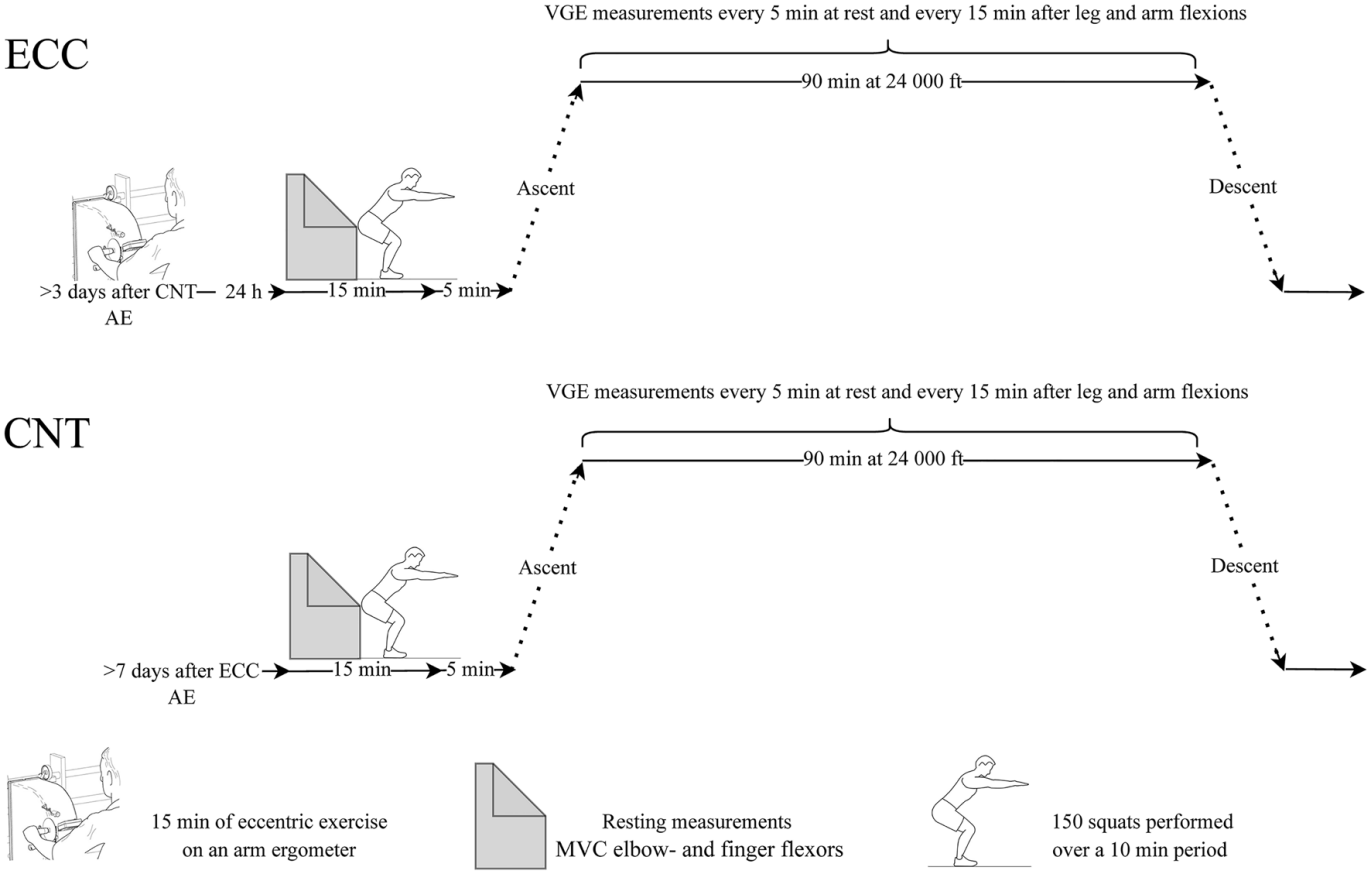
Schematic illustration of the experimental protocol. Subjects performed either eccentric exercise 24 h prior to AE (ECC) or not (CNT). Baseline measurements and unweighted squats were followed by a 90-min exposure to a simulated altitude of 24,000 ft, during which the subject was lying horizontally and performed knee and arm flex every 15 min. Abbreviations: *AE* altitude exposure, *CNT* control, *ECC* eccentric, *MVC* maximal voluntary contraction, *VGE* venous gas emboli

The experiments were carried out in the hypobaric pressure chamber (AB Motala Verkstad. 1953, Reg. nr: 20621) at the Division of Environmental Physiology, KTH Royal Institute of Technology, Stockholm. Each subject was exposed to a simulated altitude of 24,000 ft (altitude exposure; AE) on two separate occasions. In one, the AE was preceded by a bout of eccentric exercise, performed 24 h prior to the AE (ECC). The other AE served as a control (CNT). The order of the ECC and CNT trials was alternated among subjects in a balanced fashion. The trials were separated, when ECC preceded CNT by ≥ 7 days, and when CNT preceded ECC by ≥ 3 days (Fig. 1). The subjects were asked to avoid physical and sporting activities for at least 72 h prior to the two AE and prior to the eccentric exercise. The subjects were also instructed to refrain from intake of any caffeine (12 h) or alcohol-containing beverages (24 h) before AE. On the day of the first AE, the subjects’ body mass and height were measured (Vetek, Väddö, Sweden). If eccentric work had been performed 24 h prior, MVCs of the elbow and finger flexors were measured and the level of DOMS was assessed using the Borg CR10 scale (Borg 1998). Before entering the chamber, subjects performed 150 deep knee squats (unloaded) completed over a 10-min period, in an attempt to equalize the subjects’ exercise state upon exposure (Dervay et al. 2002). Thereafter, the subjects entered the chamber and were positioned on their back on a gurney. After a 5-min rest period, heart rate (HR) and arterial pressures (M3, Omron, Kyoto, Japan) were measured. Pre-gelled electrodes were attached on the thorax and neck for impedance and electrocardiography recordings (Physioflow PF07, Enduro, Manatec Biomedical Paris, France), and a pulse oximeter was placed on an index finger to allow for continuous measurement of the capillary oxyhaemoglobin saturation (SpO_2_). Just before (30–60 s) the hypobaric exposure, and throughout the exposure, subjects breathed 100% oxygen through a full-face mask (Poseidon Diving Systems AB, Göteborg, Sweden). During all exposures, the same experienced sonographer/experimenter performed VGE and DCS assessments, while also breathing 100% oxygen through a full-face mask fitted with speaking membrane, allowing for better communication with the operators outside the chamber. The experimenter completed at least 1 h of preoxygenation before any hypobaric exposure. During the experiments, the subject and inside experimenter were monitored using a video/audio system (JVC MI 5000 Victor Company, Tokyo, Japan). On each occasion, the subject was exposed to a pressure corresponding to 24,000 ft or 7315 masl (≈ 40 kPa) continuously for 90 min. The rate of pressure changes during simulated ascent/descent corresponded to 5000 ft/min. Throughout the 90-min exposure, the subjects stroke volume (SV), HR, cardiac output (CO) and SpO_2_ were monitored.

### Arm ergometer and MVC

EIMD was induced 24 h prior to the ECC altitude exposure by an eccentric exercise regimen performed on a custom-made arm ergometer, similar to that described by Elmer et al. (2013). Prior to the eccentric exercise, maximal voluntary contractions (MVC) of the elbow and finger flexors were measured bilaterally during isometric elbow flexions and handgrip contractions, respectively. The ergometer handles were set to a position allowing 90° elbow flexion and measured torque, which was then converted to force. The subject was then instructed to isometrically contract only the elbow flexor with maximal effort. The experimenter ensured that the subject did not bank when initiating the force and then maintained a maximal force for a few seconds. Grip strength was measured with a hand dynamometer (Saehan Corp., Masanhoewon-gu, South Korea) with peak force displayed until reset. MVC was performed three times in each arm and the mean value was used for further analysis. Subjects were then seated on a chair in front of the arm pedals, and the body position was adjusted in the sagittal plane (both forward/backward and upward/downward), ensuring a similar exercise position among the subjects. The hands were secured to the handles using lifting straps, to relieve the forearms and increase the work load on the bicep muscles. All subjects performed the same protocol for the arm work with 1-min eccentric work bouts repeated 15 times with 1-min rest between each bout. Time and force were set based on yet unpublished data, which showed that 15-min cranking time at 200–300 W led to EIMD. Thus, the torque output from the crank machine was adjusted prior to the first eccentric exercise bout so that it corresponded to 60% of the subject’s elbow flexor MVC (Prasartwuth et al. 2005). The subject was instructed to slow down the movements of the pedals to a cadence of 60 rpm (range 50–70 rpm). If the subject was unable to maintain the cadence, the torque output was adjusted. Throughout the exercise, the subjects were verbally encouraged by the researchers to perform maximal effort. MVC was measured in both the elbow and finger flexors and the mean value was then used for comparisons, with measurements performed at 5 min and 24 h after the eccentric work was completed. The average coefficient of variation (CV%) of the repeated MVC measurements of the elbow flexors was determined before (7%), 5-min (6%) and 24-h post-(5%) exercise.

### VGE and DCS assessment

The presence of VGE was detected from four-chamber cardiac images, using an ultrasound imaging system (CX50, Philips Ultrasound Bothell, WA, USA), equipped with a 1–5 MHz cardiac sector phased array transducer (S5-1). VGE was assessed at rest (5-min intervals), after three forceful knee flexions/extensions and after three flexions/extensions of the arms (15-min intervals). Knee and arm flexions were performed to provoke release of bubbles into the stream of mixed venous blood (Blogg and Gennser 2011; Foster and Butler 2009; Jankowski et al. 2004). Ultrasound recordings were made during ten heart beats and the prevalence of VGE was estimated during this time period. The prevalence of VGE was evaluated using the Eftedal–Brubakk (EB) six-graded scale (0–5): 0 = no visible bubbles; 1 = occasional bubbles; 2 = at least one bubble every fourth heartbeat; 3 = at least one bubble every heartbeat; 4 = at least one bubble/cm2; 5 = whiteout single bubbles cannot be discriminated (Eftedal et al. 2007). End-point criteria for the hypobaric exposures were consistent VGE score of > 3, single score of 5 and/or symptoms of DCS (e.g. joint pain). This is a validated technique and has been used repeatedly in the past to evaluate VGE load in humans both after exposure from hyperbaric chamber (Gennser et al. 2012) and during hypobaric exposures (Elia et al. 2021). VGE assessment was carried out online by the experimenter in the chamber and recordings subsequently (off-line) underwent further assessment (Elia et al. 2022). The off-line bubble rater was blinded to the conditions and the off-line assessment was used for statistical analysis. Symptoms of DCS and/or other discomfort were graded every 5 min, using a 10-point scale (Borg 1998).

### Statistical analysis

All data were statistically analysed using IBM SPSS Statistics software version 28 (IBM Corp,. Armonk, NY, USA). Kisman integrated severity score (KISS) was calculated for each subject using the bubble grades. KISS integrates scores over a period of time, and acts as a “index of severity”, giving information on the VGE load over the whole monitoring period, rather than a single point in time (Pontier and Lambrechts 2014; Elia et al. 2021). KISS was calculated according to the following formula (Pontier and Lambrechts 2014):

$$\mathrm{KISS}=\frac{100}{{4}^{\alpha }\left({t}_{n}-{t}_{1}\right)}\sum_{i=1}^{n}\frac{\left({t}_{i+1}-{t}_{i}\right)\left({d}_{i+1}^{\alpha }+{d}_{i}^{\alpha }\right)}{2},$$

where *t*_*i*_ is time of observation in minutes after reaching altitude (for time points 1 to *n*), *d*_*i*_ ultrasound score (grades 0–5) observed at time *t*_*i*_ and *α* = 3 (the parameter *α* takes into account that the bubble grade is not a linear measure of bubble quantity).

Parametrical statistical tests (ANOVA and/or paired *t* tests) were used to evaluate the significance of difference in continuous variables, i.e. HR and CO, whereas, a non-parametrical test (Wilcoxon signed-rank test) was used to evaluate differences in ordinal data, i.e. EB scores. The limit of significance was set at *P* < 0.05 (the median was used to compare ordinal data (EB scores) and mean values were used to compare continuous variables). When a chamber run had to be aborted due to DCS-related symptoms or high bubble score (*n* = 3), scores of KISS were compared up to that time.

## Results

Two exposures during the ECC condition (at min 35 and min 65) and one exposure during CNT condition (at min 80) had to be aborted due to DCS-related symptoms and/or high bubble scores. All subjects were symptom free when arriving at surface after recompression. The intra-individual comparison between exposures in these subjects was therefore performed up to the same time point as the aborted exposure. Subjects with persistent EB > 0 at ground level after the altitude exposures continued to breathe pure oxygen until bubble scores reached EB 0.

### Muscle damage

Immediately following eccentric arm cycling, MVC of the bicep flexor dropped to 63 ± 23% of the initial pre-exercise value (from 230 ± 62 N to 151 ± 86 N, *p* = 0.003) and to 79 ± 20% 24-h post-exercise (184 ± 76 N *p* = 0.042). MVC finger flexor dropped to 73 ± 22% (from 430 ± 97 N to 334 ± 160 N, *p* = 0.007) of the initial value and to 90 ± 11% (396 ± 124 N, *p* = 0.08) at 24 h. Participants reported DOMS in the regions of the biceps, triceps, trapezius, shoulders, forearms and abdomen with median 6.5 (values ranging from 4 to 9) on the Borg CR10 pain scale.

### Venous gas emboli

**Fig. 2:**
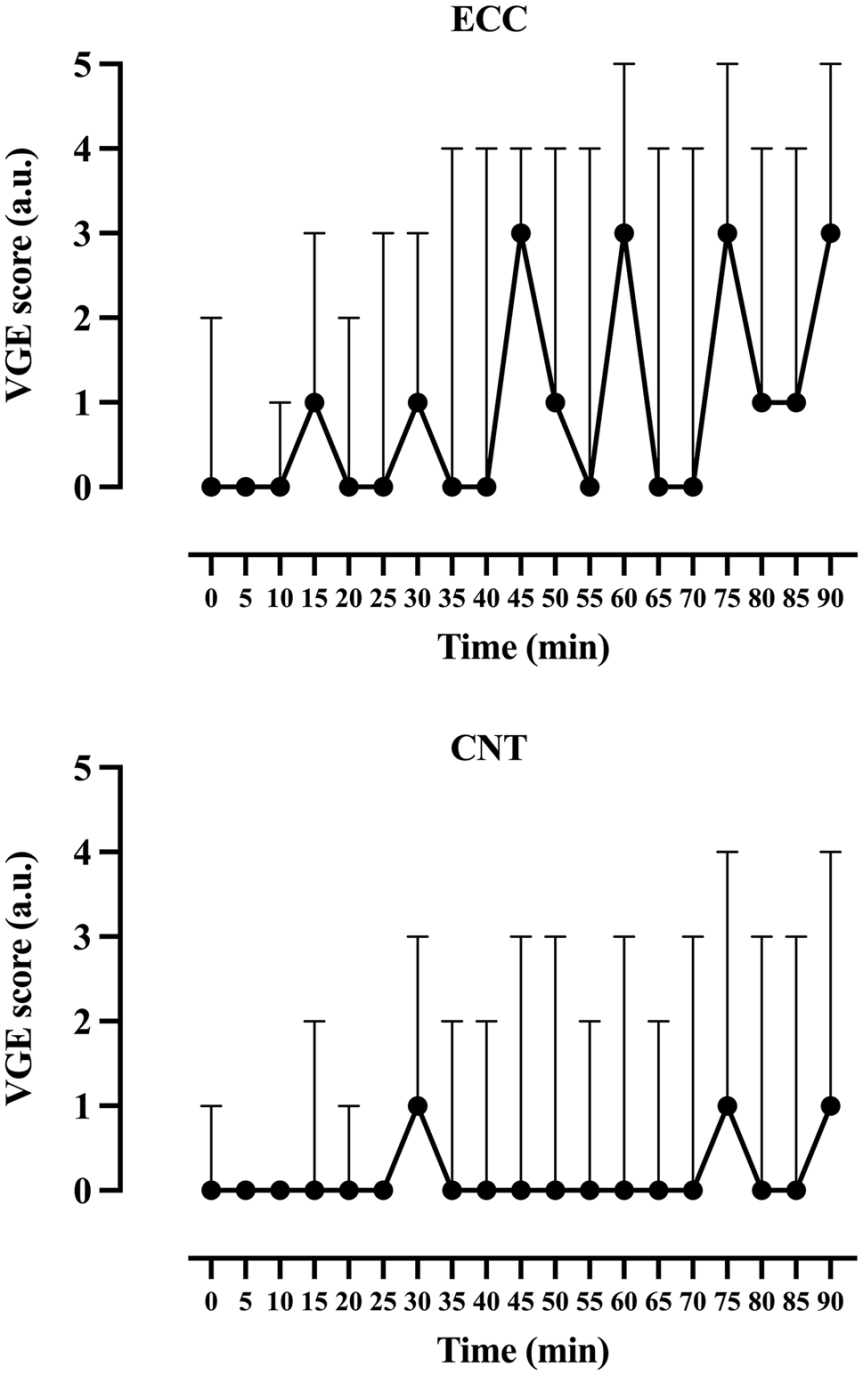
Median (line) venous gas emboli (VGE) scores in condition CNT (upper figure) and ECC (lower figure), *n* = 13. Three AE exposures (2 ECC, 1 CNT) had to be aborted. To visualize median EB scores over time, the last reported scores during rest, leg and arm flexions were used to fill out the time to 90 min. This method can be considered conservative, as bubble scores usually tend to increase in individuals with already high scores. Abbreviations: *AE* altitude exposure, *CNT* control, *EB* Efterdal–Brubakk, *ECC* eccentric, *VGE* venous gas emboli

**Fig. 3:**
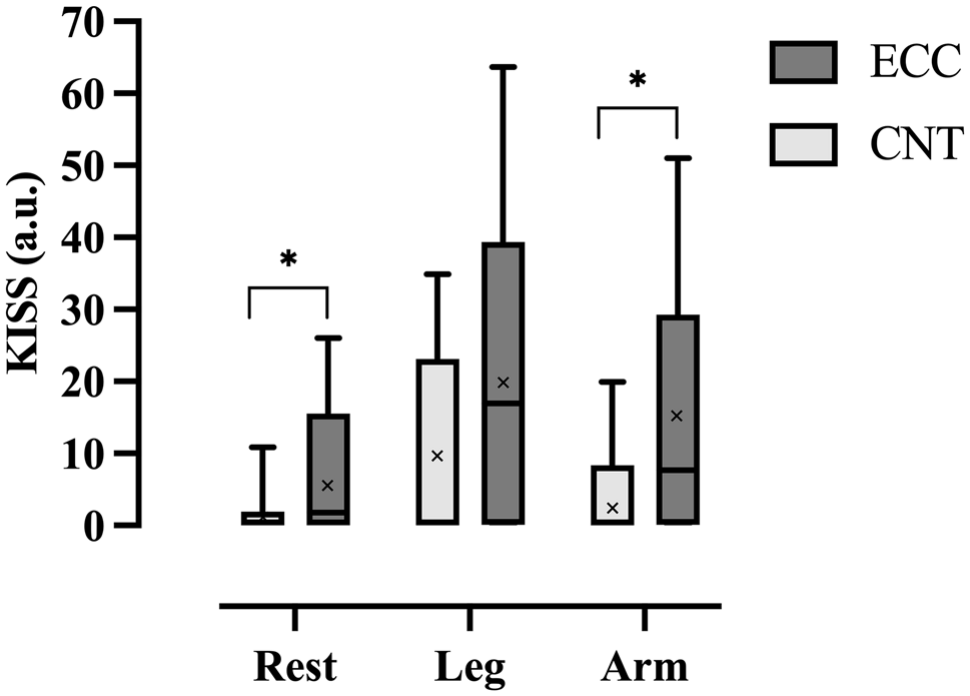
KISS shown for the different conditions. Subjects performed either eccentric exercise 24 h prior to AE (ECC) or not (CNT). The incidence of VGE was investigated both at rest, after deep knee bends and arm flex. KISS was calculated from in-flight ultrasound measurements of ten subjects. The line within the box plots represents the median, *x* represents the mean, and whiskers show high and low range. *p < 0.05. Abbreviations: *AE* altitude exposures, *CNT* control, *ECC* eccentric, *KISS* Kisman integrated severity score, *VGE* venous gas emboli

In three of the subjects, VGE were not observed in any of the altitude exposures, despite reductions in MVC and reported DOMS. In the remaining ten subjects, VGE were observed in all ECC exposures and in nine of the CNT exposures (Table 1). There was no difference in peak EB scores between subjects who carried out the altitude exposure with eccentric work before or after the control exposure (*p* = 0.589). The maximum resting, knee bends and arm-flex EB score for each subject was noted (*n* = 13) and using Wilcoxon signed-rank test, the median of these values was compared between conditions showing significantly higher EB scores in ECC than CNT, both at rest (*p* < 0.01) and after arm flex (*p* < 0.02), but not after knee bends (0.1 > *p* > 0.05) (Table 1). To visualize EB scores over time, peak median VGE scores every 5 min were plotted for the 90-min continuous exposure in both conditions (Fig. 2). Similarly, there was a significantly higher KISS in ECC than CNT at rest (6.9 ± 9.2 a.u. compared to 1.2 ± 2.3 a.u., *p* = 0.0265) and after arm flex (15.5 ± 17.3 a.u. compared to 3.8 ± 6.2 a.u., *p* = 0.029), but not after knee bends (20.1 ± 19.7 a.u. compared to 10.1 ± 13.5 a.u., *p* = 0.083*)* (Fig. 3). Additionally, we observed a difference in the time taken for the first VGE (regardless of EB score) to be detected (*p* = 0.027), with an earlier VGE manifestation being recorded during the ECC protocol (17 ± 9.2 min) compared with CNT (34.5 ± 27.4 min).

**Table 1:** Median and individual peak VGE scores (scored using the Efterdal–Brubakk 6-grade scale) collated during supine rest and following deep knee bends and arm-flex provocation

Subject	CNT			ECC		
	Rest	Leg flex	Arm flex	Rest	Leg flex	Arm flex
1	0	0	0	3	5	3
2	0	0	0	0	0	0
3	1	0	0	2	1	1
4	1	3	1	3	3	3
5	3	4	3	3	4	3
6	3	3	3	4	5	4
7	1	1	0	4	4	3
8	0	3	3	4	3	4
9	0	1	0	1	3	3
10	1	3	1	2	3	3
11	0	0	0	0	0	0
12	2	3	3	2	3	2
13	0	0	0	0	0	0
Median	1	1	0	2**	3	3*

Difference between CNT and ECC: **p < 0.01, *p < 0.02

### Cardiovascular variables

There were no significant differences between conditions in any of the cardiovascular variables, either at rest [HR, 63 ± 9 (CNT), 61 ± 9 (ECC) *p* = 0.199; CO, 4.86 ± 0.92 (CNT), 5.54 ± 1.68 (ECC), *p* = 0.372], during the knee-bend provocations [HR, 74 ± 9 (CNT), 71 ± 10 (ECC) *p* = 0.292; CO, 6.24 ± 1.34 (CNT), 6.97 ± 2.24 (ECC), *p* = 0.326], or during the arm-flex provocations [HR, 66 ± 8 (CNT), 63 ± 9 (ECC) *p* = 0.363; CO, 5.35 ± 1.28 (CNT), 6.08 ± 1.77 (ECC), *p* = 0.149].

## Discussion

The aim of this study was to investigate whether, and to what degree, exercise-induced muscle damage will increase decompression strain, as indicated by increased vascular bubble formation, during a hypobaric decompression. The results demonstrated that muscle damage induced by eccentric arm work 24 h prior to ascent from sea level to 24,000 ft, significantly increased the formation of decompression bubbles during rest and arm-flex provocation, but not during knee-bend provocation. Furthermore, there was a significantly earlier onset of first VGE in the ECC exposures.

Strength loss after eccentric exercise is considered to be a valid and reliable marker of muscle damage (Warren et al. 1999). Thus, our finding that the eccentric exercise bout reduced MVC immediately and 24 h after exercise (reduction in MVC to 63% and 79% from baseline, respectively) is in line with other studies on eccentric exercise of the elbow flexors (Jones et al. 1987; Newham et al. 1987; Stauber et al. 1990) and implies that the exercise bout indeed induced microscopic muscle injury. One of the cardinal symptoms associated with tissue injury and inflammation is pain, and hence, the subjects reported delayed-onset muscle soreness in several upper body muscles after the exercise, which corroborates the notion of EIMD and suggests that it was not limited to the biceps brachii muscles. It is well documented that the overstretching of sarcomeres during high-intensity eccentric muscle actions results not merely in a drop in MVC, but also in a local inflammatory response characterized by DOMS (Proske and Morgan 2001). The choice to perform exercise 24 h prior to a hypobaric exposure was based on the fact that most literature on eccentric exercise has shown mechanical (Proske and Morgan 2001; Fridén et al. 1981), inflammatory (MacIntyre et al. 1995) and microvascular (Hotta et al. 2018) changes within this time window. Whether or not the decompression stress is maximum during that point in time has to be explored further.

Seven days might not have been enough to completely restore the EIMD; however, it appears from indirect markers of muscle damage (i.e. drop in strength, soreness and biomarkers) that the affected muscle will almost, if not completely, recover by day 7 after a maximal eccentric bout (MacIntyre et al. 1995; Jones et al. 1987). In the present study, the interval between the exposures carried out in the order CNT–ECC was (mean (range)) 12 (5–28) days and for ECC–CNT order it was 16 (8–28) days. In addition, the orderly sequence CNT/ECC exposure was alternated in a balanced fashion, securing that any carryover effect between trials was balanced between conditions.

That both the peak and time-integrated VGE scores were higher in the ECC than the CNT altitude exposure suggests that EIMD increased the formation of VGE during decompression. There appears to be no similar controlled experimental study on humans reported in the literature, but a possible relationship between prior musculoskeletal injury and susceptibility to DCS has been discussed in the literature concerning hypobaric decompression (Fryer 1969; Adler 1964). Thompson et al. tested the relationship between old injury and DCS susceptibility by collecting data on serious injuries of the extremities (i.e. fractures, sprains, etc.) in flight crews, prior to their first hypobaric exposure. In a group of 1220 men, 327 gave a history of a fracture or other injuries to a limb (Thompson et al. 1944). Upon decompression to altitude, merely 18% of these men developed pain in proximity to the location of the old injury, and the authors concluded that there was little, if any, relationship between old injury and the localization of DCS. It is, however, difficult to interpret the results of this study (Thompson et al. 1944), since there was no account of the DCS incidence in the control group (893 men without a previous serious limb injury). In the same report, it was concluded that DCS pain tended to localize more frequently in a region which had been subjected to a recent minor injury. Subjects given a blow on the tibia prior to being exposed to 35,000 ft, experienced increased incidence of bends in the same region (17% vs 8% in the control group) (Thompson et al. 1944). Houston et al. studied 1538 man-ascents to altitude and determined the coincidence of bends at the site of previous injuries to be 7.2%, which was considered a significant relationship (Houston et al. 1944). Notably, even though the occurrence of DCS correlates to the prevalence of decompression-induced VGE, comparison of results must be done with caution, between studies using DCS (as in the aforementioned studies) vs VGE (present study) as the main effect variable.

Light aerobic exercise in combination with preoxygenation prior to hypobaric decompression has been shown to significantly reduce the incidence of DCS compared to resting preoxygenation (Webb et al. 1996; Hankins et al. 2000; Loftin et al. 1997). Since the elimination and uptake of nitrogen is a perfusion-limited process, this additive effect is attributed to augmented denitrogenation resulting from increased blood flow through the exercising muscles (Webb et al. 1996). Indeed, moderate concentric arm or leg exercise performed during decompression from hyperbaric pressure has also been found to reduce VGE (Jankowski et al. 1997, 2004). However, exercise without preoxygenation (150 knee flexes over 10 min) just prior to depressurization led to significant increases in bubble formation (Dervay et al. 2002). This effect is probably unrelated to muscle damage and more likely, as the authors discuss, is due to generation of new micronuclei or enlargement of existing micronuclei. At altitude, deep knee bends followed by weighted arm extensions have been found to decrease time to maximum VGE score and to increase the incidence of DCS compared to sedentary conditions (Krutz and Dixon 1987). Similarly, isometric and dynamic arm and leg exercises induce DCS at otherwise considered symptom-free altitudes (Pilmanis et al. 1999). The increase in VGE and DCS is probably due to production of new bubbles, similar to what is observed following knee flexes prior to decompression.

Regarding exercise prior to hyperbaric exposure, it has been shown to reduce (Jurd et al. 2011; Blatteau et al. 2005; Dujic et al. 2004) or have no effect on circulating bubbles (Gennser et al. 2012) upon decompression. The results seem to vary depending on the type, duration and intensity of the exercise, as well as on how long before decompression the exercise was performed. The protocols used in these studies consisted of aerobic exercise (running or cycling), dominated by concentric muclse contractions, known to induce considerably less muscle damage than eccentric actions (Lavender and Nosaka 2006; Peñailillo et al. 2013). Presumably, these exercise regimens did not inflict substantial muscle damage, but rather, may have served to, possibly by way of increased peripheral blood flow, remove precursor micronuclei adhering to the endothelial walls (Vann et al. 1980; Arieli and Marmur 2017).

Also, with regard to studies in experimental animals, information is scarce and equivocal on effects of muscle damage on the susceptibility to decompression stress. Thus, Jørgensen et al., showed that eccentric exercise performed by rats prior to simulated diving had no effect on bubble formation (Jørgensen et al. 2015, 2013). Harvey, on the other hand, found that, in cats, skeletal muscle injury inflicted shortly prior to hypobaric decompression increased the presence of circulating VGE (Harvey 1945). The reason for the discrepancy between the results of the two animal studies may well be that the intervention by Jörgensen et al. was considerably milder than the one by Harvey. Jørgensen et al. used downhill running to induce eccentric exercise and hence muscle damage in the rats, whereas, Harvey inflicted damage in the thigh muscles by applying local pressure (squeezing them). The difference between present results and those by Jørgensen, remains to be settled. At least in humans, downhill running is associated with considerably less muscle damage than high-force eccentric actions (Clarkson and Hubal 2002).

The question arises as to the mechanisms underlying the augmented VGE prevalence in the ECC trial. It is rather well established that, in vivo, decompression VGE, and in particular altitude-induced VGE, originate from already pre-existing micronuclei attached to the endothelial walls (Christman et al. 1986; Lee et al. 1993; Vann et al. 1980; Arieli and Marmur 2011), since spontaneous (de novo) formation of gas bubbles in a solution with dissolved gas requires a marked supersaturation corresponding to a pressure differential of about 10.0 MPa (1000 msw) (Jones et al. 1999).

Our observation that there was a significant difference of bubble load in the ECC condition during rest indicates a humoral effect. However, the significant difference after arm flex but not leg flexion suggests that the general increase in VGE results from increased bubble formation locally in muscle-damaged regions. This assumption, as well as the extent to which the importance of total affected muscle mass, will have to be confirmed in future studies. Peak EB and KISS scores after knee flexions were the same in both conditions in five participants, resulting in no significant difference between conditions (Table 1 and Fig. 3). During deep knee bends, big muscle groups of the legs and the trunk are activated, and consequently, knee bends are usually followed by high VGE scores during prolonged exposure to 24,000 ft (Ånell et al. 2020; Elia et al. 2021). VGE scores in the CNT condition were similar to those seen in previous studies with a similar protocol of 24,000 ft for 60–90 min (Ånell et al. 2019, 2020; Elia et al. 2021), giving rise to peak bubble scores of 2–3 on the EB scale. There was also a substantially earlier occurrence of first VGE, regardless of grade, in the ECC than in the CNT condition. Conversely, Elia et al. found a significant latency before the first VGE was detected in a preconditioning study consisting of 30-min whole-body vibration prior to hypobaric decompression. They also found lower maximum VGE scores in the pre-exposure vibration condition than in the control condition (Elia et al. 2021). It thus seems that, depending on whether an intervention generates more or fewer bubbles, it also affects the onset of VGE.

Assuming that, following eccentric exercise, VGE is increased locally in the damaged muscle, then one might, albeit speculatively, consider a few mechanisms that might contribute to local formation of micronuclei. Firstly, analyses of biopsy samples after eccentric exercise have shown possible damage and disturbance to capillaries (Stauber et al. 1990) and in vitro experiments suggest microvascular hyperpermeability during EIMD (Hotta et al. 2018). Conceivably, the ECC-induced increase of VGE could be due to increased leakage of micronuclei from affected muscle to vessels, perhaps in combination with increased intramuscular formation of microbubbles due to inertial cavitation induced by the traction of sarcomeres (Kim et al. 2021). In addition, early in the recovery period, neutrophiles propagate an inflammatory response by secretion of cytokines. In the following days, both proinflammatory and anti-inflammatory cytokines are secreted, acting to clear damaged tissue and initiate regeneration of muscle fibres (Peake et al. 2017; Moldoveanu et al. 2001). Possibly, these molecules could coat the microbubbles, reducing the surface tension of the bubble by reducing the attractive forces between the gas molecules in the bubble. This would lead to a greater influx of nitrogen in existing microbubbles. Lastly, in vitro experiments have shown that on a completely hydrophobic surface (e.g. glass), the zero-contact angle means that no bubble can stick or remain, whereas with any positive contact angle gas nuclei can stick and be stable. EIMD might change the local structure on endothelia tissue, by increased areas of depressions and cavity, creating more positive contact angles for micronuclei to adhere. Nuclei might then enlarge and break away from the surface as a bubble, which continue to grow.

In summary, eccentric exercise induces muscle damage and the literature is unanimous in that this leads to local disruption of tissue and capillaries, with subsequent systemic inflammation. We hypothesize that this will increase the presence of gaseous micronuclei. When and for how long it is possible to observe increased bubble production following eccentric exercise, and whether the increase in VGE occurrence is due to local or humoral effects, remain to be settled.

### Methodological considerations

The study was not performed it in a double- or single-blinded fashion and hence was limited by the fact that not only the subjects, but also the experimenters were aware of at what time point they had performed exercise. This was deemed a necessary safety precaution, to avoid confusing DOMS with DCS. On the other hand, the same subjects were used in both conditions, allowing for intra-individual comparison. The same ultrasound operator was present in all chamber runs and the scoring was verified after each experiment by another experienced sonographer.

### Implications and conclusions

EIMD increases the presence of circulating VGE; it is however unclear if it also increases the risk of DCS, although a large number of bubbles and high grades predispose to DCS (Francis and Mitchell 2003). To date, there is no recommendation regarding flying or diving after injury or EIMD. However, the results in the present study makes this question worthy of being explored further. Specifically, it will be interesting to examine whether, and to what extent, addition of muscle groups would further increase/advance VGE production. Additionally, it would be of interest to explore whether EIMD following simulated dive in a hyperbaric chamber increases circulating VGE. If so, caution might be needed both when planning diving and flying decompression procedures.

In conclusion, the present study demonstrates that 15 min of eccentric upper body exercise 24 h prior to decompression significantly increases the prevalence of VGE and advances the generation of VGE during a continuous 90-min exposure at 24,000 ft compared to a control flight in the same individuals.

## Data Availability

Data supporting the study findings may be requested from the corresponding author (F.G), but are not publicly available since they contain information that could compromise the privacy of the research participants.

## Abbreviations

ANOVA: Analysis of variance
AE: Altitude exposure
CNT: Control
DCS: Decompression sickness
DOMS: Delayed-onset muscle soreness
EB: Eftedal–Brubakk
ECC: Eccentric
EIMD: Exercise-induced muscle damage
FMD: Flow-mediated dilation
KISS: Kisman integrated severity score
MVC: Maximal voluntary contraction
VGE: Venous gas emboli
